# Bifunctional Catalysts Synthesized from Hierarchical Materials and Highly Dispersed Metallic Particles: A New Approach

**DOI:** 10.1021/acscentsci.4c01486

**Published:** 2024-10-11

**Authors:** Juan Antonio Cecilia, Antonio M. Pérez-Merchán, Benjamín Torres-Olea

**Affiliations:** † Departamento de Química, Inorgánica, Cristalografría y Mineralogía, Facultad de Ciencias, Universidad de Málaga, Campus de Teatinos s/n, 29017 Málaga, Spain; ‡ Instituto de Investigación en Biorrefinerías “I3B”, Facultad de Ciencias, Universidad de Jaén, Universidad de Granada, Universidad de Sevilla, Universidad de Málaga, Campus de Teatinos s/n, 29071 Málaga, Spain

Throughout the 20th century, the field of catalysis has developed enormously due to scientific and industrial advances.^1^ As a result, the design of catalysts has been optimized to obtain maximum yields under milder conditions. The first catalytic studies were carried out in gaseous or liquid media, where a substance was added to accelerate or inhibit a chemical process. More recently, the focus has shifted to the dispersion of the active phase, aiming to increase the catalytically active surface area, reduce the amount of active phase materials used, and save costs. With the development of microporous and mesoporous materials with ordered structures and modulable pore sizes,^2,3^ the next challenge for the scientific community was designing catalysts with pore sizes adapted to the dimensions of the reactant and product molecules in such a way that the active phase could be homogeneously dispersed in the porous material. Traditional methods for dispersing the metallic phase include incipient wetness impregnation and precipitation. However, these methodologies often produce particles with highly heterogeneous crystal sizes, and in most cases, the active phase appears on the external surface of the support material.^4,5^ Another recent trend in catalyst synthesis is the development of multifunctional catalysts. These materials are designed to combine catalysts with hydrogenating, acidic, basic, oxidizing, or reducing characteristics, enabling consecutive reactions to occur in a single step, thereby reducing costs.^6,7^

**Figure 1:**
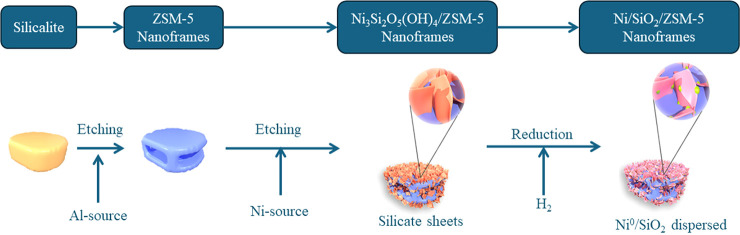
Synthetic mechanism of Ni/SiO_2_/ZSM-5 nanoframes.

Considering the scientific community’s interest in this area, Tian et al. developed bifunctional catalysts with both acid and metal centers, as detailed in their paper titled “Construction of metal/zeolite hybrid nanoframe reactors via in situ-kinetics transformations”, published in *ACS Central Science*.^6^ The authors focused on achieving small, homogeneous metallic particles within a microporous structure to prevent pore blockage and the resulting diffusion problems caused by metal particles blocking the channels. They selected silicalite-1 nanocrystals, a well-described material, as a template for the synthesis of ZSM-5 nanoframes. This procedure was performed in two steps. In the first step, silicalite-1 templates were etched through the recrystallization of ZSM-5 around silicalite in alkaline media, leading to frame-like nanoarchitectures with hierarchical porosity. In the second step, the ZSM-5 nanoframes were enveloped with layered Ni_3_Si_2_O_5_(OH)_4_ nanosheets, after which both Ni^2+^-species and silica were etched in the ZSM-5 nanoframes. Finally, the Ni^2+^-species were reduced to metallic Ni^0^ under a H_2_-flow, resulting in well-dispersed particles across the surface of the ZSM-5 structures, with homogeneous crystals averaging 4.5 nm in size (Figure 1).^6^

**Figure 2:**
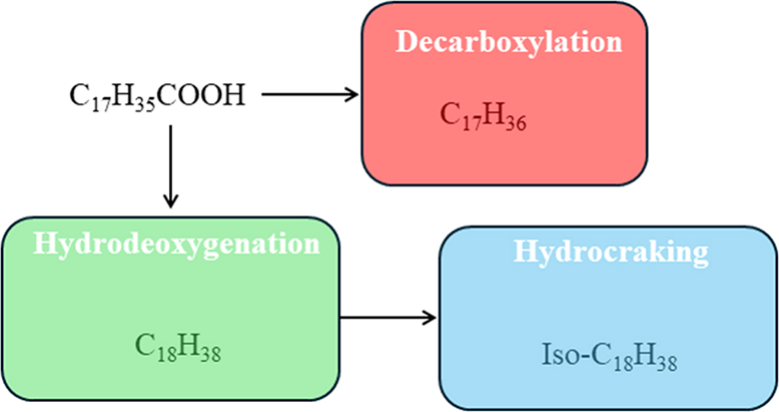
Reaction scheme for the hydrodeoxygenation of stearic acid.

The authors then utilized these metal/zeolite hybrid nanoframes in the hydrodeoxygenation (HDO) of stearic acid to obtain diesel derivatives, although this type of catalyst can be extrapolated to other processes where bifunctional catalysts are required (Figure 2). In this reaction, the presence of acid sites promotes hydroisomerization and hydrocracking, while the metallic sites are responsible for the HDO.^9^ The catalytic results indicate that the porosity and acidity of the zeolite, as well as the dispersion of the metallic sites, significantly impact the catalytic behavior for obtaining diesel derivatives, confirming that this synthetic approach is well-suited for designing catalysts with tunable pore sizes and acidity, and highly dispersed metallic phases.^1^

With this methodology, the Si/Al molar ratio can be modulated,^8^ allowing for control over the amount of acid sites in the catalysts. In the same way, this approach can be used to modulate Lewis and Brönsted acid sites by selectively blocking certain acid sites or through dealumination or desilication processes. Such high versatility makes it possible to tailor ZSM-5 nanoframes for a wide range of reactions, such as hydrocracking, hydroalkylation, isomerization, and dehydration, among others. Additionally, the incorporation of metallic species into the ZSM-5 nanoframes promotes the dispersion of small, homogeneous metallic particles. Tian et al. also pointed out that it would be possible to introduce several transition metals, such as Ni, Co, Fe, and even bimetallic phases, all with small, homogeneous crystal sizes. These features highlight the broad versatility of this methodology for synthesizing bifunctional catalysts.^1^
